# Taking Cold

**Published:** 1870-07

**Authors:** 


### TAKING COLD.

Many a fatal form of sickness is brought into action by a slight cold. Many a friend has been lost to us in that way, who but for a trivial exposure at a critical condition of the system at the time, might have still been alive. Many a time the same exposure, an hour sooner or later, would have had no ill effect whatever. If for example, you enter a vehicle in a profuse perspiration, and sit near an open window, with a strong draught of air, a cold is inevitable, but if you had taken the same seat an hour before, not having exercised so as to cause perspiration, no damage would have resulted. A man can never become so hardy but that he will take cold if he cools off too soon, or if he allows himself to remain uncomfortably cold in a still position until he becomes chilled through and through. It is well therefore to know and remember a few of the things which are likely to give a cold.

1. Going to sleep in the day-time, especially without any covering, even in midsummer, with the ordinary clothing on, because during sleep the circulation is less active than when awake, and is not sufficiently active to repel cold

2. Sitting in a room where there is a fire, with the back toward the window, because there is a draught made by the cold air driving into the room through the crevices.

3. Sitting still with damp feet even for half an hour has often induced fatal consequences.

4. Failing to change the clothing after it has become damp.

5. Putting on a cold pair of shoes or slippers immediately after having come in from a walk.

6. Coming into a parlor or otherwise unused room in spring or fall, when there is no fire, while the exercise in the pleasant, balmy air from without has caused some perspiration. A gentleman should keep his hat on, and a lady should keep in motion while waiting for the person visited to come in. It is not unusual to keep parties waiting in a cold parlor so long as to get completely chilled.

7. After entering a room without fire from a brisk walk, none of the outer clothing should be removed for several minutes, unless busied on the feet, so as to keep up the circulation to a point equal to what it was while out of doors.

8. Prominent persons have brought on serious and even fatal sickness in foreign lands by visiting picture-galleries or rooms of other art, especially in Rome, which never have fire in them, and almost always have a sepulchral dampness, rather pleasant on first entering, but which rapidly diminishes the heat of the body, inducing chills, pneumonia, and death. Hence it is wise, on entering a building or room without fire from the warm sunshine from without, to put on an additional garment, or at least draw those already on closer around you, yet it is quite common to see persons take off the hat, lay aside the gloves, remove the shawl, the moment they come in from the street.

9. In riding in vehicles it is safer to raise the front glasses instead of those at the side or in the rear.

10. Going to the street door in cold weather to have the last words with a friend, often protracted until the whole frame is chilled, has brought serious sickness to many. But this whole class of cases is comprehended in three injunctions: —

1. Avoid draughts under all circumstances.
2. Cool off very slowly after all forms of exercise.
3. Never remain still for an instant under a feeling of chilliness.

